# The association between exposure to a radio campaign on nutrition and mothers’ nutrition- and health-related attitudes and minimal acceptable diet of children 6–36 months old: a quasi-experimental trial

**DOI:** 10.1017/S1368980024001319

**Published:** 2024-10-21

**Authors:** Bernard Appiah, Mahama Saaka, George Appiah, Lucy Asamoah-Akuoko, Elfreda Samman, Laura Forastiere, Brenda AZ Abu, Abena A Yeboah-Banin, Irene A Kretchy, Freda D Ntiful, Christiana NA Nsiah-Asamoah, Md Koushik Ahmed, Christopher R France

**Affiliations:** 1 Research Program on Health Communication and Public Engagement (H-COPE), Department of Public Health, Falk College, Syracuse University, 150 Crouse Dr, 435A White Hall, Syracuse, NY 13244, USA; 2 Centre for Science and Health Communication, PMB M71, Accra, Ghana; 3 Department of Nutritional Sciences, School of Allied Health Sciences, University for Development Studies, P O Box 1883, Tamale, Ghana; 4 National Blood Service Ghana, Research and Development, Korle-Bu, P.O. Box KB 78, Accra, Ghana; 5 Department of Health Behavior, School of Public Health, Texas A&M University, College Station, Texas, TX, USA; 6 Laura Forastiere, Department of Biostatistics, Yale School of Public Health, New Haven, CT, USA; 7 Wegmans School of Health and Nutrition, College of Health Sciences and Technology, Rochester Institute of Technology, Rochester, NY, USA; 8 Department of Communication Studies, School of Information and Communication Studies, University of Ghana, Legon, Accra, Ghana; 9 Department of Pharmacy Practice and Clinical Pharmacy, School of Pharmacy, University of Ghana, Legon, Accra, Ghana; 10 Department of Nutrition and Dietetics, School of Biomedical and Allied Health Sciences, College of Health Sciences, University of Ghana, Legon, Accra, Ghana; 11 Department of Clinical Nutrition and Dietetics, University of Cape Coast, Cape Coast, Ghana; 12 Department of Psychology, Ohio University, Athens, OH, USA

**Keywords:** Radio campaign, Nutrition education, Minimum acceptable diet, Health and nutrition attitudes

## Abstract

**Objective:**

To evaluate the effectiveness of a radio campaign involving serial 10-minute drama episodes, 10-minute on air discussion of each episode by trained community health workers and 30-minute phone-ins from listeners in improving mothers’ nutrition- and health-related attitudes (HNRAs) and children’s minimum acceptable diet (MAD).

**Design:**

A two-arm quasi-experimental trial with a pre-post design was used to quantify the effect of a radio campaign on nutrition before and immediately after the 6-month intervention. Difference-in-difference (DID) analysis was performed to assess the intervention’s effect.

**Setting:**

Saboba district (intervention) and Central Gonja (comparison district) of northern region of Ghana.

**Participants:**

At baseline, a total of 598 mothers with children aged 6–22 months were randomly selected from the intervention (*n* 298) and control (*n* 300) districts. At endline (6 months post-intervention), 252 mother–child dyads in the intervention district and 275 mother–child dyads in the control district were followed up.

**Results:**

The radio campaign was significantly and positively associated with a change in health- and nutrition-related attitudes (HNRA) over time, with DID in mean attitudes significantly improving more over time in the intervention district than the control (DID = 1·398, *P* < 0·001). Also, the prevalence of MAD over time in the intervention district was significantly higher than the control district (DID = 16·1 percentage points, *P* = 0·02) in the presence of food insecurity.

**Conclusions:**

The study indicates that a radio campaign on nutrition is associated with improved mothers’ HNRA and children’s MAD. Communication interventions on child nutrition targeting low-resource settings should consider this innovative approach.

Child malnutrition including stunting and wasting remains a serious public health challenge globally, especially in many low- and middle-income countries. According to the 2021 Global Nutrition report, most countries are not on track to achieve the 2025 global targets set for infant and young child nutrition indicators such as childhood stunting, childhood overweight, exclusive breastfeeding and childhood wasting^(1)^. For example, only thirty-five countries are on course to achieve exclusive breast-feeding as a target, with thirty-three either making no progress or having worsening exclusive breast-feeding rates.

Childhood malnutrition, which often results from suboptimal feeding practices, is associated with negative health outcomes such as poor physical growth and increased risk for non-communicable diseases later in life^(2,3)^. Given the negative consequences of childhood malnutrition, there is an urgent need to generate effective, evidence-based interventions^(2,4–6)^.

Radio and television may offer a cost-effective approach to reduce childhood malnutrition. For example, a review of studies on the effectiveness of mass media interventions in improving child survival in low- and middle-income countries identified a total of 111 campaign evaluations, with fourteen of them focusing on child nutrition^(7)^. The nutrition campaigns, which focused primarily on priority populations, namely women of reproductive age, caregivers with children of age 6–24 months old and health care workers, were found to have positive effects on self-reported behaviours such as minimum dietary diversity^(8,9)^ and consumption of foods rich in iron^(9)^ and vitamin A^(8,10)^. Of the fourteen studies, the most frequently used communication channels were radio (79 %) and television (57 %). The interventions typically took the form of unique radio ads (57 %) or television ads (43 %); however, other formats were also noted including radio drama, radio songs and longer television programmes ^(7)^.

More recently, a systematic review of the effectiveness of mass media and nutrition education interventions on infant and young child feeding practices identified eighteen studies globally, but only one involved radio alone^(11)^. Most interventions included a mix of mass media and other educational approaches. The radio only intervention, which was conducted in Burkina Faso, focused on timely initiation of breast-feeding, exclusive breast-feeding at 0–5 months, complementary feeding 6–11 months and growth monitoring for children of age 6–23 months old^(12)^. The intervention, which was known as ‘Saturation+’, involved airing 1-minute radio spots at least ten times per day, and a 2-hour program that aired five nights per week with a 10-minute live radio drama acted on location by local actors. The rest of the program was taken up by news, music and discussion.^(12)^ Saturation+ did not find statistically significant differences in dietary practices between intervention and control clusters. Despite the absence of a significant effect for this particular study, radio remains the most accessible and pervasive media in most of Africa’s rural poor communities, making it indispensable as a potential form of behaviour change communication^(13)^. One potential enhancement that may increase the likelihood of success of radio campaigns is careful consideration of the delivery agent. In many rural communities, community health workers are key players in promoting child health. For example, a recent trial empowered health extension workers in Ethiopia to educate rural mothers on nutrition and found that the intervention significantly reduced the rate of infant stunting by 7·5 percentage points (26·5 % *v*. 34 %, RR = 0·68; 95 % CI: 0·47, 0·98)^(14)^. Thus, integration of community health workers into radio campaigns may be a novel way to improve maternal nutrition and health-related attitudes and in turn promote childhood nutrition.

Accordingly, the present study reports on the evaluation of an innovative radio campaign, called ‘10 + 10 + 30’, which included three components: a 10-minute serial radio drama on child nutrition and other health issues, a 10-minute on-air discussion of the drama by community health workers and a 30-minute phone-in opportunity for listeners of the radio programme to ask questions of the community health workers. The goals of the radio campaign intervention were to improve mothers’ health and nutrition-related attitudes, and in so doing promote a healthy diet among their children.

## Methods

### Setting

The study was conducted in two northern districts of Ghana, namely Saboba (intervention site) and Central Gonja (control site). Both districts have predominantly rural communities, with Soboba being 90·6 % rural^(15)^ and Central Gonja being 80 % rural^(16)^. Up to 93 % of households in Saboba district are engaged in smallholder agriculture and 97·7 % are involved in crop farming ^(15)^. Similarly, 74·2 % of households in the Central Gonja district engage in smallholder agriculture and 92·3 % are involved in crop farming^(16)^.

### Intervention

The 6-month radio campaign was titled ‘My family health and nutrition time’, which was translated to Lekwakwa language in Saboba as ‘Mamaal aalaafee ni tijikanyaan aayonn’. The intervention was developed and implemented based on the theory of planned behavior. According to this theory, three main factors – attitudes, subjective norms and perceived behavioural control – influence intention to adopt a particular health behaviour of interest (e.g. mothers providing children with diversified diets)^(17)^. The design and implementation of the 10 + 10 + 30 radio intervention integrated the constructs of this theory. For example, 10 + 10 + 30 intervention was aimed to increase mother’s intention to adopt the behaviour of interest by promoting local, healthy diets for children and letting mothers know of the benefits of adequate complementary feeding. The intervention also targeted how the peers and spouses could positively influence the behaviour of the priority population – mothers – regarding the recommended practices. Also, it aimed to make mothers believe that they had control over being able to implement the recommended practices such as giving their children diverse diets and feeding them appropriately.

The radio campaign was implemented in Saboba from July 2018 to January 2019 on Radio Gaakii of Saboba on Mondays and Thursdays in the evening from 19.30 to 20.20. The chosen days were non-market days so that many of the market women and farmers would be at home at that time, and therefore, more likely to listen to the radio campaign, with their families.

The intervention involved twenty-one serial drama episodes with titles including a four-star diet. A four-star diet is a food group classification used in Ghana for nutrition education. It is a balanced diet that includes foods from the four major food groups. Each food group is considered a star, and thus mothers are encouraged to ensure that their children earn four ‘stars’ to ensure that the children get varied diets.

Other drama episodes addressed micronutrients, nutrition in pregnancy, benefits of antenatal care, men as partners, breast-feeding, childhood immunisation and growth monitoring (Table 1). Immediately after each 10-minute drama episode, a journalist served as a moderator and posed preset questions about the drama to two trained community health workers including a community health nurse. The discussion with the community health workers lasted 10 min, after which phone lines were activated for listeners to call in. Each 10-minute radio drama episode that aired on Monday was repeated on Thursday. However, the 10-minute live discussion of the drama and 30-minute live listener phone-ins were different for each day. In other words, of the 10 + 10 + 30 components, only the drama was repeated.

**Table 1:** The number of phone-ins from listeners of a radio campaign on health and nutrition implemented in northern region, Ghana, July 2018 to January 2019, based on topics

Week	Drama episode/Topic^*^	Number of phone-ins from listeners
1	Healthy lifestyle (first program)	6
2	Sanitation (first program)	12
3	Food hygiene (First program)	27
4	Cultural belief – nutrition of mother and children (first program)	9
5	Adolescent health (first program)	23
6	Teenage pregnancy (first program)	n/a
7	Four-star diet (first program)	15
8	Micronutrients (first program)	n/a
9	Nutrition in pregnancy (first program)	n/a
10	Benefits of antenatal care (first program)	6
11	Men as partners (first program)	27
12	Malaria control in pregnancy-LLIN, IPTp (first program)	n/a
13	Prevention of anaemia in pregnancy (first program)	14
14	Facility delivery (first program)	24
15	Initiation of breast-feeding (first program)	31
16	Exclusive breast-feeding (first program)	22
17	Complementary feeding (first program)	28
18	Childhood immunisation (first program)	n/a
19	Post-natal care (first program)	14
20	Birth spacing (first program)	12
21	Childhood welfare clinic – deworming, vitamin A, LLIN, Growth monitoring (first program)	n/a

* Each drama episode was aired twice in a week.

LLIN = long-lasting insecticidal net. IPTp = Intermittent preventive treatment of malaria in pregnancy.

Of the 24 weeks of the intervention phase, twenty one had 10 + 10 + 30 radio intervention, which was created to aid engagement with listeners to facilitate increased uptake of health and nutritional information for child nutrition and health in general. The first week was an introduction of the radio campaign, and the last two weeks were used to recap key lessons.

#### Study design

The study used a quasi-experimental design with non-randomised intervention and control groups: an intervention district that received the radio transmissions and a control district that did not have access to the radio transmission. The two districts in Northern Ghana were selected because they were similar with respect to their demographics and setting, yet far enough apart (approximately 261 km) that it was not possible for participants in the control district to listen to radio transmissions from the intervention district. The transmission of the radio station reached the entire intervention district. Moreover, respondents from the two trial districts spoke different local languages, and thus even in the unlikely event of the intervention reaching the comparison district, residents in the comparison district would not have been able to understand the intervention.

#### Sample size calculation

The sample size was estimated by assuming power of 80 %, in a two-tailed statistical significance of 5 %, 0·35 difference in mean dietary diversity score between intervention and control groups and 10 % attrition rate. Using the sample size formula for unmatched cluster-randomised trials^(18)^, 300 eligible households in the intervention and 300 eligible households in the control were needed (twenty households from each of fifteen clusters per arm).

#### Sample selection

In the intervention district, fifteen clusters/communities were randomly selected using probability proportionate to size. This was repeated for the control district. A sample of twenty households with eligible mothers/caretaker and children were randomly selected per cluster, resulting in a total of 598 households (intervention: 298; control: 300). Two questionnaires from the intervention district had incomplete data. With the help of community volunteers, households in each community were serially numbered to obtain the rough total number. A systematic random sampling was used to select the households from the list.

All the households in each cluster were serially numbered. To obtain the sampling interval, the total number of households in a cluster was divided by the sample size of 20. The first household was then randomly selected by picking any number within the sample interval. Subsequent selections were made by adding the sampling interval to the selected number to locate the next household to visit. The next household was selected using the systematic sampling procedure if the selected household did not have an eligible respondent. This process continued until the required sample size was obtained, ensuring that only one eligible participant was selected from each household. In situations where more than one eligible child was available, simple random selection through the lottery method was applied to select only one.

#### Data collection

Trained data collectors conducted interviewer-administered baseline surveys in the local languages spoken in the intervention and control districts. Baseline data were collected in April 2018 3 months before the campaign began, and final survey data were collected in February 2019 (the campaign ended in January 2019).

The survey included items on socio-demographic characteristics, attitudes related to infant, child and young feeding practices, dietary intake of children by asking mothers to recall food items their children had eaten in the past 24 h and food security based on three items about whether the household experienced any difficulty accessing food in the past 4 weeks, and if so how often this happened. The responses to the food insecurity questions were ‘No (did not happen)’, ‘Yes (did happen) rarely (1–2 times)’, ‘sometimes (3–10 times)’ and ‘often (more than 10 times)’. The three three items were taken from the Household Food Insecurity Access Scale that describe food access and how often households have difficulty accessing food^(19)^.

Two main means of measuring exposure to radio content were used: unaided recall and aided recall. In the unaided recall, respondents were asked to indicate their frequency of listening to radio, with answer options ‘at least once a week’, ‘less than once a week (once in a while)’ and ‘not at all’. In the aided recall, respondents were asked a) to indicate whether they had listened to radio drama on nutrition, agriculture and/or public health within the past 3 months and b) the frequency of listening to the radio drama, with two answer options: at least once a week and less than once a week (once in a while).

#### Data analysis

*χ* ^2^ test was used to assess the differences in socio-demographic characteristics, frequency of listening to radio and exposure to radio drama on nutrition between the intervention and control districts.

The mother’s education was categorised as none (no formal education), low (Primary & Junior High School) and high (at least Senior High School). However, in the difference-in-difference (DID) analysis, mother’s education was re-coded as a binary variable: at least Senior High School and below Senior High School. The reference education category was education below Senior High School.

To analyse food insecurity, the responses to three subset questions from the Household Food Insecurity Access Scale that pertain to insufficient food quantities in the past 4 weeks were used^(19)^. A code of ‘0’ was used for households that replied ‘No’ occurrence of food insecurity question. A frequency response of ‘rarely’ was coded as ‘1’, and a frequency response of ‘often’ was coded as ‘2’. For the DID analysis, the answers to the three items were regrouped into two (0/1 or none/mild and 2 or moderate/severe household food insecurity). The reference category was none or mild food insecurity.

We focused on two main outcomes: minimum acceptable diet (MAD) and health- and nutrition-related attitudes (HNRAs).

For MAD, two main indicators – minimum meal frequency and minimum dietary diversity – were computed from mothers’ 24-hour dietary recall of their children’s diet, with a focus on eight food groups: (i) grains, roots and tubers; (ii) legumes and nuts; (iii) dairy products; (iv) flesh foods (meat, poultry and fish); (v) eggs; (vi) vitamin A-rich fruits and vegetables; (vii) other fruits and vegetables and (viii) breastmilk.

MAD is based on both minimum meal frequency and minimum dietary diversity. Minimum meal frequency and minimum dietary diversity were assessed according to WHO guidelines.^(4)^ Minimum meal frequency was achieved if the child received complementary foods at least the minimum recommended number of times in 24 h. For breastfed children, the minimum recommended number of times are two times (for children aged 6–8 months) and three times (for children aged 9–11 months or 12–23 months)^(4)^. The minimum meal frequency was 4 for non-breastfed children. A child aged 6–23 months was deemed to have met minimum dietary diversity if the mother indicated that the child had eaten from at least five food groups out of eight food groups in the past 24 h^(4)^ A child who met both minimum meal frequency and minimum dietary diversity was regarded to have met MAD (indicated as 1) and one not meeting both minimum meal frequency and minimum dietary diversity as having failed to meet minimal acceptable diet (indicated as 0).

HNRAs were estimated from ten key behaviours/statements related to appropriate child feeding. For example, one of the statements was ‘Give your children a variety of foods for healthy growth’. HNRAs were measured on a three-point Likert scale with three response options: agree (scored as 2), neutral (scored as 1) and disagree (scored as 0). The scores from the ten items were added to get a total score. The scores ≥ median score of questions related to HNRA were considered high whereas those less than the median score were considered low. However, for the DID modelling, HNRA as an outcome was treated as a continuous variable, whereas MAD was treated as a categorical variable.

Using DID approach, the differences in outcomes of HNRAs and MAD were estimated between the intervention and control groups at baseline and endline. The DID approach is a statistical method used to estimate treatment effects comparing the pre- and posttreatment differences in the outcome variables of a treatment and a control group. It relies on the parallel trend assumption, that is, it assumes that without the intervention the trend of the outcome over time would have been parallel between the intervention and the control group. Thus, by comparing key outcomes at baseline in the district that received radio campaign (a) and final evaluation survey data (b), and key outcomes in the control district at baseline (c) and final evaluation survey (d), one could calculate DID as (b-a)-(d-c) while controlling for the differences in baseline characteristics and general development over time^(20)^. DID was used to analyse the outcomes, MAD and HNRAs, by generating linear probability models, or linear models, respectively. DID is usually implemented as an interaction term between time and treatment group dummy variables in a regression model as shown:

Y = β0 + β1 * [Time] + β2 * [Intervention] + *β*3 * [Time * Intervention] + β4 * [Covariates] + ε^(21)^

The statistical analyses were performed using the Statistical Package for the Social Sciences, version 23.0 (SPSS), and using the complex survey module to account for the complex design of cluster sampling. A *P* value of < 0·05 was considered statistically significant.

## Results

Listeners were interested in the intervention radio show, and there were 270 phone ins. Episodes on food hygiene, men as partners, breast-feeding and complementary feeding received high phone-ins from listeners compared with other topics (Table 1).

### Socio-demographic characteristics of study participants at baseline

As shown in Table 2, the mean age of the respondents in the intervention communities was significantly higher than that of the control communities (29·2 years ± 5·8 *v.* 27·9 years ± 5·7). Table 2 compares the details of the socio-demographic characteristics of the respondents in the study groups at baseline. The two study arms differed in terms of characteristics such as radio listening frequency, religion, educational level and type of occupation. These baseline differentials were adjusted for in establishing relationships between outcomes and exposure.

**Table 2:** Demographic and other characteristics of the participants (*n* 598); 6–23-month-old children and their mothers, at baseline by intervention and control arm, and comparison of difference between arms, northern region, Ghana, April 2018

	Total (*n* 598)	Intervention (*n* 298)	Control (*n* 300)	
Participants characteristics	%	%	%	*P* ^*^
Child’s sex				
Male	48·3	49·3	47·3	0·6
Female	51·7	50·7	52·7	
Child’s age (months)				
6–11	44·1	34·6	53·7	0·001
12–23	55·9	65·4	46·3	
Mother’s age (years)				
18–34	83·4	82·9	84·0	0·7
35^+^	16·6	17·1	16·0	
Maternal education				
None (No formal education)	63·7	61·1	66·3	0·02
Low (Primary & Junior High School)	26·6	25·8	27·3	
High (at least Senior High School)	9·7	13·1	6·3	
Marital status				
Not married	2·3	3·7	1·0	0·03
Married	97·7	96·3	99·0	
Religion				
Islam	55·3	12·8	97·7	0·001
Christianity	34·3	66·4	2·3	
Others	10·4	20·8	0	
Mother’s occupation classification				
Agriculture	65·6	72·1	59·0	0·003
Office-based professionals	4·3	4·7	4·0	
Non-office-based professionals	21·9	17·4	26·3	
None	8·2	5·7	10·7	
Wealth index classification^†^				
Low	37·1	42·8	31·8	0·006
High	62·9	57·2	68·2	
Household radio access				
Yes	62·4	74·2	50·7	0·001
No	37·6	25·8	49·3	
Frequency of radio listening				
Not at all	32·9	20·1	45·7	0·001
Less than once a week	15·2	22·8	7·7	
At least once a week	51·8	57·0	46·7	

* Statistics using χ2 test.

† Household wealth index was calculated by generating a score for number of ‘Yes’ responses to nine questions about household commodities: radio, television, mobile telephone, refrigerator, bicycle, motorcycle, car or truck, tricycle, and animal-drawn cart.

### Exposure to listening to radio campaign on nutrition and health at endline

At endline, a significantly greater proportion of respondents listened to the radio campaign using drama, discussion and phone-in on nutrition and health at least once a week in the intervention communities (87·4 %) than in the comparison communities (59·3 %) (χ2 = 32·5, *P* < 0·001). Although the 10 + 10 + 30 radio campaign was not aired in the comparison district, 59·3 % of respondents indicated that they listened to it at endline.

### The association between listening to a radio campaign and mothers’ health- and nutrition-related attitudes

Using the DID analysis and controlling for potential covariates, 10 + 10 + 30 radio campaign was significantly associated with improved HNRA over time (Table 3). As shown by the interaction term in the regression model, the mean DID in mean HNRA was significantly higher over time in the intervention district than the control (DID = 1·398, *P* < 0·001).

**Table 3:** The association between listening to a radio campaign on nutrition and health and mothers’ health- and nutrition-related attitudes*, northern region, Ghana, February 2019

Model	Unstandardised coefficients		*P* ^†^	95·0 % CI for *β*
	β	Std. Error		Lower bound, upper bound
Time of survey (Endline)	0·761	0·36	**0·03**	0·06, 1·46
Intervention arm of study (Treatment)	−1·493	0·26	**< 0·001**	−1·99, –0·99
Child’s age in months	−0·049	0·02	**0·02**	−0·09, –0·007
Mother’s educational level (at least Senior High School)^‡^	0·291	0·12	**0·02**	0·05, 0·53
Listening to nutrition and health campaign on radio at least once a week^‡‡^	0·421	0·21	**0·04**	0·02, 0·82
Interaction term (treatment × time)^‡‡‡^	1·398	0·37	**< 0·001**	0·68, 2·11

* Health and nutrition-related attitudes were created from ten statements:

1. Start complementary feeding at 6 months; not earlier, not later.

2. Give your children a variety of foods for healthy growth.

3. Green leafy vegetables are rich in substances that help the body to make blood for children and adults.

4. To prevent anaemia, give your children fish and meat frequently in sufficient amount.

5. Add fats and oils to your children’s food for strength and vitality.

6. Give porridge that are thick enough to stay on the spoon for better nutrition and growth.

7. Make foods thicker, mashed or chopped fine as your baby gets older.

8. Always remember to wash your hands with soap and water before handling your child’s food.

9. Keep your cooking utensils clean and safe from germs.

10. Give your children vitamin A and deworming tablets along with supplements.

Also, health- and nutrition-related attitudes (HNRAs) were measured on a three-point Likert scale with three response options: agree (scored as 2), neutral (scored as 1) and disagree (scored as 0). The scores from the ten items were added to get a total score. HNRA were treated as a continuous variable.

† Significant *P* values are indicated in bold.

‡ In this model, mother’s highest level of education (at least Senior High School) was compared with the lowest level of education (lower than Senior High School): the reference category.

‡‡ The reference category for listening to nutrition and health campaign on radio was not listening to radio intervention.

‡‡‡ The interaction term in the DID model represents changes in the main outcome variable (mothers’ health- and nutrition-related attitudes) in the intervention arm compared with the control over time (from baseline to endline).

The results showed that the mean HNRAs was significantly lower by 1·493 (Std. Error = 0·26) in the intervention group, compared with the comparison group at baseline (Table 3). However, the expected mean change in HNRAs with the passage of time as shown by the survey time term in the comparison district in the absence of the actual intervention (i.e. the counterfactual) was significant (*P* = 0·03). Compared with respondents who did not listen to the intervention, those who listened to it at least once a week had a mean health and nutrition-related attitude which was significantly higher (*P* = 0·04) (Table 3).

Other factors that associated significantly with HNRA included mother’s educational level and age of child (Table 3). The mean health and nutrition-related attitudes was higher among women who attained highest educational level (Beta = 0·09, *P* = 0·02), compared with their counterparts who had no formal education.

### The association between listening to a radio campaign and children’s minimum acceptable diet

As shown in Table 4, over the intervention period of 6 months, there was significantly higher prevalence of MAD in the intervention communities compared with the control communities (DID = 16·3 percentage points, *P* = 0·02). Compared with respondents who did not listen to the intervention, those who listened to the intervention at least once a week had a prevalence of MAD, which was significantly higher by 0·10 standard units (Beta = 0·10, *P* = 0·008).

**Table 4:** The association between listening to a radio campaign on health and nutrition, and children’s minimum acceptable diet*, northern region, Ghana, February 2019

Model^‡^	Unstandardised coefficients		*P* ^†^	95·0 % CI for *β*
	β	Std. error		Lower bound, upper bound
Time of survey (Endline)	0·048	0·068	0·48	−0·09, 0·18
Intervention arm of study (Treatment)	0·211	0·050	**< 0·001**	0·113, 0·31
Moderate or severe household hunger^‡^	−0·094	0·045	**0·04**	−0·18, −0·006
Child age in months	0·010	0·004	**0·02**	0·002, 0·017
Listening to nutrition drama on radio at least once a week^‡‡^	0·103	0·039	**0·008**	0·03, 0·18
Interaction term (treatment × time)^‡‡‡^	0·163	0·070	**0·02**	0·025, 0·30

* Minimum acceptable diet is based on both minimum meal frequency and minimum dietary diversity. Minimum meal frequency and minimum dietary diversity were assessed according to WHO guidelines. Minimum meal frequency was achieved if the child received complementary foods at least the minimum recommended number of times in 24 h. For breastfed children, the minimum recommended number of times are two times (for children aged 6–8 months) and three times (for children aged 9–11 months or 12–23 months). The minimum meal frequency was 4 for non-breastfed children. A child aged 6–23 months was deemed to have met minimum dietary diversity if the mother indicated that the child had eaten from at least five food groups out of eight food groups in the past 24 h. A child who met both minimum meal frequency and minimum dietary diversity was regarded to have met minimum acceptable diet (indicated as 1) and one not meeting both minimum meal frequency and minimum dietary diversity as having failed to meet minimal acceptable diet (indicated as 0).

† Significant *P* values are indicated in bold.

‡ The Household Food Insecurity (HFI) was quantified using three items from the Household Food Insecurity Access Scale that describe food access and how often households had difficulty accessing food^(19)^. A code of ‘0’ was used for households that replied ‘No’ occurrence of food insecurity question. A frequency response of ‘rarely’ was coded as ‘1’, and a frequency response of ‘often’ was coded as ‘2’. For the DID model, the answers to the three items were regrouped into two (0/1 or none/mild and 2 or moderate/severe household food insecurity). The reference category was none or mild food insecurity.

‡‡ The reference category for listening to nutrition and health campaign on radio was not listening to radio intervention.

‡‡‡ The interaction term in the DID model represents changes in the main outcome variable (minimal acceptable diet) in the intervention arm compared with the control over time (from baseline to endline).

## Discussion

The study assessed whether a 6-month radio campaign involving a 10-minute drama, a 10-minute discussion of the drama by community health workers, and a 30-minute phone-in from listeners could influence mothers’ health and nutrition-related attitudes and the prevalence of a child’s MAD. The key findings were that, after controlling for potential covariates, the health and nutrition radio drama series was associated with more positive maternal health and nutrition-related attitude, and a higher a prevalence of MAD of children, in the intervention district relative to the control district. In low- and middle-income countries, systematic reviews on the effectiveness of mass media and nutrition education demonstrate a positive impact on the prevalence of MAD.^(11,22–24)^

The results of the current study are also consistent with nonradio-based behavior change communication interventions showing improved child dietary diversity in Kenya^(25)^, Malawi^(26)^ and Uganda^(27)^ and MAD in Ethiopia^(28)^. In terms of the positive effect of the nutrition radio campaign on attitudes of mothers, the results mirror those found in non-radio educational interventions in Ethiopia^(29)^, a radio intervention in Ghana that involved radio drama, discussion, phone-in from listeners and radio spots^(30)^ and an intervention comprising scripted messages delivered by nurses and via radio in Mexico^(8)^.

In the present study, children whose mothers had formal education of at least Senior High School were more likely to be fed a MAD. Similar results from cross-sectional studies including Demographic and Health Surveys have been reported from several countries including Ethiopia^(31,32)^. A possible explanation for the association may be related to the fact that mothers with higher education levels have greater job opportunities and higher decision-making power, which in turn facilitate improvement in the use of health services^(33)^.

The prevalence of MAD was significantly higher in households which experienced little to no household hunger than in households that reported moderate or severe household hunger. Similar findings have been reported in Northern Ghana and elsewhere^(34–36)^. The results of the present analyses suggest that advocating for policies that aim to promote household food security could be a pathway to achieving child feeding adequacy.

Similar to prior studies that assessed media exposure and the prevalence of children’s minimal acceptable diet^(37,38)^, in the present study, children whose mothers listened to the radio at least once a week, were more likely to have higher prevalence of MAD than children of mothers who listened to the radio less than once a week or not at all. This might be because frequent listening to the radio might enhance the mother’s awareness and capacity of feeding a MAD to their children. For example, a study conducted in Ethiopia showed that mothers’ exposure to the media significantly increased the odds of children achieving MAD by 1·7 (*P* = 0·002) although the type of mass media was not indicated^(37)^. The nature of the intervention in the current study might have also increased knowledge of mothers. For example, the aspect of the 30-minute phone-in from listeners might have given some mothers the opportunity to ask questions to clarify certain misconceptions about the nutritional needs of growing children. Also, it was interesting that among the episodes that received high number of callers were initiation of breastfeeding (thirty-one listeners), complementary feeding (twenty-eight listeners) and men as partners (seventy-seven listeners).

Although the current study did not include interpersonal communication, the findings closely resemble a nutrition trial conducted in Nigeria using radio and community health workers providing individual and group counselling sessions^(39)^. The study in Nigeria led to increased immediate breast-feeding (50·9 % in intervention group compared with 28·3 % in control group). Given that the current study empowered community health workers to use radio for promoting childhood nutrition, the findings could bolster interventions that such as the one in Nigeria that aim to use community health workers counsel mothers of children on nutrition.

To the best of our knowledge, this study is the first to assess radio only intervention with drama, discussion and phone-in but lacking radio spots on mothers’ HNRAs and children’s minimal acceptable diet. Many mass media interventions implemented to influence nutrition had other mass media channels including television or other educational approaches such as in-person counselling. Unlike the present intervention, two radio only interventions on nutrition included radio spots^(12,30)^. The simplicity of 10 + 10 + 30 radio campaign on nutrition could make it more cost effective in low- and middle-income countries particularly in rural populations. This is because most rural households in low- and middle-income countries may often get health and nutrition information primarily from community radio. With airtime cost for television being high when compared with community radio (a type of radio station owned by communities rather than the government or business people), and rural residents unlikely to read newspapers, a simple 10 + 10 + 30 radio campaign could be cost-effective in improving childhood nutrition in low- and middle-income countries.

Nevertheless, our study has some limitations. The evaluation survey did not include an item for respondents to indicate whether they were among the callers into the radio programme. Such an indicator might have helped to identify those who were more likely engaged in listening to the programme. Also, MAD was measured through self-report, which could lead to recall bias. Moreover, although we used two approaches for measuring media exposure (unaided recall in terms of frequency of listening to radio in a typical week) and aided recall (in terms of frequency of listening to radio drama on nutrition, health or agriculture), using recognition might have resulted in improved exposure measurement^(40)^. A brief audio from the drama with major characters could have been played for respondents to identify whether they are familiar with the radio characters. Using such a method for measuring media exposure might have boosted the ability to easily distinguish the 10 + 10 + 30 from other radio programmes. For example, some households in the comparison communities reported listening to the radio campaign on nutrition and health. This was confirmed at a seminar held to disseminate the results of the study to key stakeholders at the regional level. It came to light that the World Food Programme implemented a nutrition project, including using radio to increase awareness of the project at the comparison district at the same time as our intervention. While the World Food Programme’s project did not have a radio drama, respondents in the comparison district might have confused that intervention with our unique 10 + 10 + 30 intervention.

Future studies should consider alternative measurements of media exposure. Finally, the World Food Programme’s nutrition project implemented in the comparison district by the World Food Programme at the same time as our intervention might have diluted the effect of the 10 + 10 + 30 intervention on child nutrition.

### Conclusion

The results of the present study provide preliminary evidence to support the potential benefit of promoting maternal and child health in low-resource, rural communities using a radio campaign supported by community health workers. Although further testing is necessary using larger samples that are carefully matched on a range of socio-demographic characteristics, the current findings complement existing evidence from other countries to suggest that radio is a promising medium to enhance rural health based on its pervasiveness, accessibility and ability to engage communities with expert health messaging.
